# Supplementary material for: Distinct Urinary Metabolite Signatures Mirror In Vivo Oxidative Stress-Related Radiation Responses in Mice
Source: Antioxidants (Basel). 2024 Dec 27;14(1):24. doi: 10.3390/antiox14010024 (PMC11763242; doi:10.3390/antiox14010024)
Supplement: Supplementary file 1 [file antioxidants-14-00024-s001.zip › Figures S1 and S2.pptx]

## Slide 1
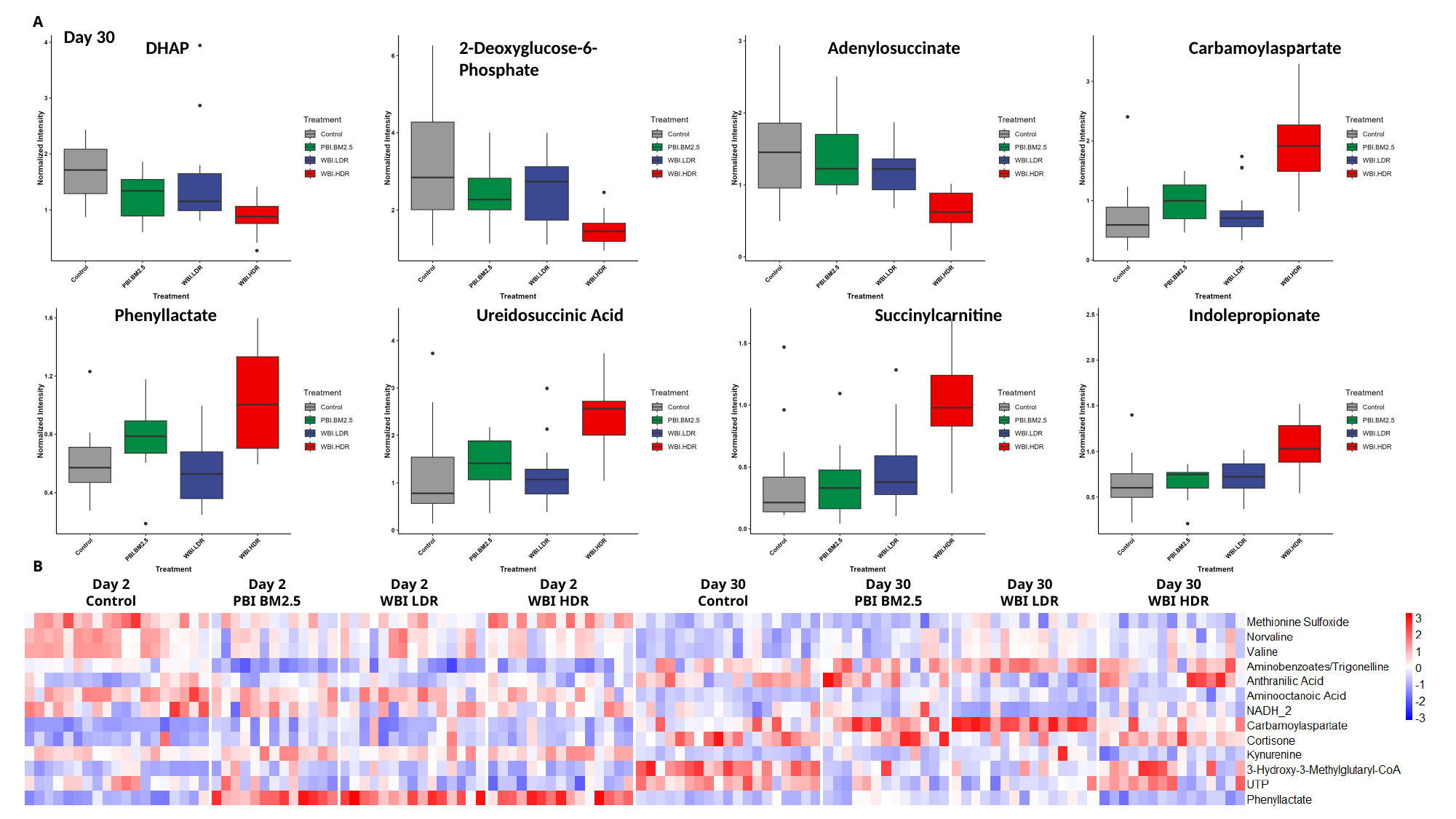

A
Day 30
DHAP
2-Deoxyglucose-6-Phosphate
Adenylosuccinate
Carbamoylaspartate
Phenyllactate
Ureidosuccinic Acid
Succinylcarnitine
Indolepropionate
B
Day 2
Control
Day 2
PBI BM2.5
Day 2
WBI LDR
Day 2
WBI HDR
Day 30
Control
Day 30
PBI BM2.5
Day 30
WBI LDR
Day 30
WBI HDR

## Slide 2
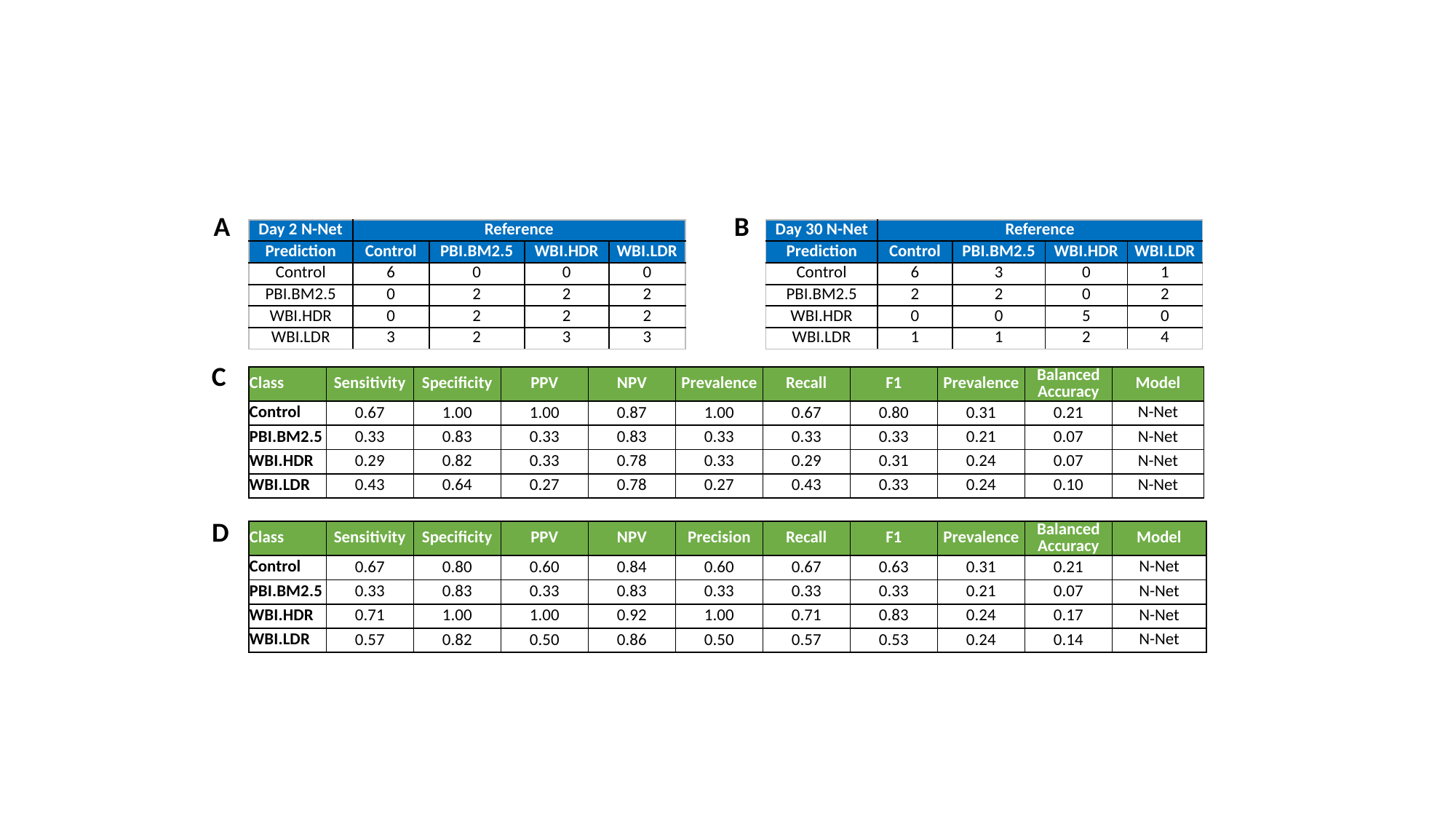

A
B
| Day 2 N-Net | Reference | | | |
| --- | --- | --- | --- | --- |
| Prediction | Control | PBI.BM2.5 | WBI.HDR | WBI.LDR |
| Control | 6 | 0 | 0 | 0 |
| PBI.BM2.5 | 0 | 2 | 2 | 2 |
| WBI.HDR | 0 | 2 | 2 | 2 |
| WBI.LDR | 3 | 2 | 3 | 3 |
| Day 30 N-Net | Reference | | | |
| --- | --- | --- | --- | --- |
| Prediction | Control | PBI.BM2.5 | WBI.HDR | WBI.LDR |
| Control | 6 | 3 | 0 | 1 |
| PBI.BM2.5 | 2 | 2 | 0 | 2 |
| WBI.HDR | 0 | 0 | 5 | 0 |
| WBI.LDR | 1 | 1 | 2 | 4 |
C
| Class | Sensitivity | Specificity | PPV | NPV | Prevalence | Recall | F1 | Prevalence | Balanced Accuracy | Model |
| --- | --- | --- | --- | --- | --- | --- | --- | --- | --- | --- |
| Control | 0.67 | 1.00 | 1.00 | 0.87 | 1.00 | 0.67 | 0.80 | 0.31 | 0.21 | N-Net |
| PBI.BM2.5 | 0.33 | 0.83 | 0.33 | 0.83 | 0.33 | 0.33 | 0.33 | 0.21 | 0.07 | N-Net |
| WBI.HDR | 0.29 | 0.82 | 0.33 | 0.78 | 0.33 | 0.29 | 0.31 | 0.24 | 0.07 | N-Net |
| WBI.LDR | 0.43 | 0.64 | 0.27 | 0.78 | 0.27 | 0.43 | 0.33 | 0.24 | 0.10 | N-Net |
D
| Class | Sensitivity | Specificity | PPV | NPV | Precision | Recall | F1 | Prevalence | Balanced Accuracy | Model |
| --- | --- | --- | --- | --- | --- | --- | --- | --- | --- | --- |
| Control | 0.67 | 0.80 | 0.60 | 0.84 | 0.60 | 0.67 | 0.63 | 0.31 | 0.21 | N-Net |
| PBI.BM2.5 | 0.33 | 0.83 | 0.33 | 0.83 | 0.33 | 0.33 | 0.33 | 0.21 | 0.07 | N-Net |
| WBI.HDR | 0.71 | 1.00 | 1.00 | 0.92 | 1.00 | 0.71 | 0.83 | 0.24 | 0.17 | N-Net |
| WBI.LDR | 0.57 | 0.82 | 0.50 | 0.86 | 0.50 | 0.57 | 0.53 | 0.24 | 0.14 | N-Net |
